# DIR-Net: Deep Residual Polar Decoding Network Based on Information Refinement

**DOI:** 10.3390/e24121809

**Published:** 2022-12-12

**Authors:** Bixue Song, Yongxin Feng, Yang Wang

**Affiliations:** School of Information Science and Engineering, Shenyang Ligong University, Shenyang 110159, China

**Keywords:** polar codes, deep learning, DIR-Net, denoising subnetwork, decoding subnetwork, attention mechanism

## Abstract

Polar codes are closer to the Shannon limit with lower complexity in coding and decoding. As traditional decoding techniques suffer from high latency and low throughput, with the development of deep learning technology, some deep learning-based decoding methods have been proposed to solve these problems. Usually, the deep neural network is treated as a black box and learns to map the polar codes with noise to the original information code directly. In fact, it is difficult for the network to distinguish between valid and interfering information, which leads to limited BER performance. In this paper, a deep residual network based on information refinement (DIR-NET) is proposed for decoding polar-coded short packets. The proposed method works to fully distinguish the effective and interference information in the codewords, thus obtaining a lower bit error rate. To achieve this goal, we design a two-stage decoding network, including a denoising subnetwork and decoding subnetwork. This structure can further improve the accuracy of the decoding method. Furthermore, we construct the whole network solely on the basis of the attention mechanism. It has a stronger information extraction ability than the traditional neural network structure. Benefiting from cascaded attention modules, information can be filtered and refined step-by-step, thus obtaining a low bit error rate. The simulation results show that DIR-Net outperforms existing decoding methods in terms of BER performance under both AWGN channels and flat fading channels.

## 1. Introduction

Polar codes are a forward error correction coding method applied in communication systems based on the theory of channel polarization and are closer to the Shannon limit than other channel coding methods [1]. Researchers have paid attention to them for their lower complexity of coding and decoding. In recent years, they have emerged as a hot topic for academic research, especially for their successful application in fifth-generation mobile communication (5G) [2,3]. As a result, this study focuses on how to further enhance the performance of the decoder.

The conventional decoding techniques can be generally classified as successive elimination (SC) [1] and belief propagation (BP) algorithms [2], in addition to enhanced techniques based on the abovementioned schemes. One of them, the SC algorithm, has low computational complexity but suffers from high latency and low throughput. To address this issue, some studies concentrated on low-latency SC decoding techniques [4,5], which combine the stability of recognition-based techniques with the dynamism of check-based techniques. In addition, some studies have been presented to improve error correction performance [6,7,8,9,10,11,12]. While all of these are serial techniques, which suffer from random node errors, some parallel decoding techniques, including the BP algorithm [13,14], have been proposed to address this issue. Despite the improved error performance of the BP algorithm, it has high complexity due to iterative computation.

Deep learning has been demonstrated to be a potent technique for enhancing the speed and performance of algorithms in the domains of computer vision and natural language processing. Recently, the technology has also been applied to communication systems, including channel coding. Deep neural networks have been proposed to assist conventional decoding in the area of polar decoding, such as lowering latency [15,16,17,18,19,20], extracting sequence features [21,22,23], reducing the memory overhead [24], and learning the noise correlation [25]. The construction of deep learning decoding networks has recently caught the attention of some researchers. A deep feed-forward neural network was introduced in [26] to directly decode the input polar codes. A fully connected network was proposed in [27] to decode polar-coded short packets over a flat fading channel. Wei et al. demonstrated that the neural network could learn the entire coding structure by comparing the three network structures of MLP, CNN, and RNN under the same conditions [28]. To improve the SNR of received symbols, a denoiser based on residual learning was introduced before the neural network decoder (NND) in [29], which was characterized as a residual neural network decoder (RNND). In [30], a double long short-term memory (DLSTM) neural network was proposed to improve the performance of short block length by clipping frozen bits to enhance prediction accuracy. Deep learning-based decoders perform better than traditional decoders [31]. However, most decoding technologies treat deep neural networks as black boxes, where the decoding network consists of only a few fully connected or convolutional layers that take polar codes as inputs to the network and output decoded symbols directly. These techniques assume that all information, including noise and some other information, positively contributes to the decoding.

In this paper, a novel deep-learning framework is proposed for polar decoding to improve accuracy. Unlike traditional deep learning decoders which use all input information indiscriminately for decoding, a deep residual network based on information refinement (DIR-Net) with attention mechanism is designed to refine the information step-by-step, and it achieves high-quality decoding. We utilize a large number of attention modules in the designed decoding network in order to better distinguish between valid and disturbing information. The proposed DIR-Net is divided into two stages, the denoising stage and the decoding stage. In the former stage, a denoising subnetwork (DN-SBN) is constructed to remove the noise in the received codewords. Here, we utilize 1D convolution layers with residual connection to construct the base subnetwork. Then, we add an attention module after each convolution block to separate the valid information from the noise. In the decoding stage, a decoding subnetwork (DC-SBN) with a fully connectivity and attention mechanism is constructed. The designed attention module can refine the input information and preserve only the effective information for decoding. By cascading these attention modules, the performance of the decoder can be gradually improved. Experimental results show that our proposed DIR-Net outperforms existing decoding methods and obtains state-of-the-art performance.

The main contributions of this paper are summarized as follows:

(1) We design a novel information optimization module for polar decoding based on the attention mechanism. This module can avoid the interference of invalid information during the decoding.
(2) With the designed information optimization module as the core, we construct an advanced deep learning decoding network. To our knowledge, this is the first time that the attention modules are used completely to construct a decoding network for polar codes. The network refines information step-by-step by cascading multiple attention modules and exhibits excellent decoding performance.
(3) We evaluated the effectiveness of the proposed decoding network in the Gaussian channel and the Rayleigh fading channel, respectively. The experimental results demonstrate that our proposed decoding network can effectively suppress the interfering information and achieve high BER performance in both channels.

The remainder of the paper proceeds as follows: the decoding method of polar codes is first introduced in the next section. Then, the details of the proposed method are presented. In Section 4, the experimental results are shown to demonstrate the effectiveness of our method. We summarize our study in the last section.

## 2. Background

### 2.1. Polar Code

A polar code of length *N* with *K* information bits can be expressed as (*N*, *K*), where *K* channels are used to transmit information bits, and *N*–*K* channels are used to transmit frozen bits. The encoding process of polar codes can be described as follows:

(1) $$x_{1}^{N}=u_{1}^{N}G_{N},$$

where $x_{1}^{N}=\left\{x_{1},x_{2},\ldots ,x_{n}\right\}\;$represents the codeword bits, $u_{1}^{N}$ represents the source bits, $G_{N}=B_{N}F^{⊗n}$ is the generator matrix. $B_{N}$ is the bit-reversal permutation matrix, and $F^{⊗n}$ denotes the n-th Kronecker power, with

(2) $$F=\left[\begin{array}{cc} 1 & 0\\ 1 & 1 \end{array}\right].$$

### 2.2. Neural Network Decoder

Unlike the traditional decoding schemes which decode the codewords with analytical or iterative methods, deep learning decoding methods implement decoding tasks by deep neural networks. The decoding network is trained offline with huge amounts of data and used online in the real world. To obtain a high-quality decoder, large amounts of training data need to be collected first. The training data consist of noisy polar codes and the original decoded signal. The former is the input of the decoding network, and the latter is the corresponding label. The data can be collected in the communication system with polar codes or by computer simulation. The latter is the method most used in the literature. In general, training data are specified with a code length *k*, and there are *n* code words $X=\left\{x_{1},x_{2},\ldots ,x_{n}\right\}\;$under the code length requirements, where each code word is $x_{n}=\left\{x_{n}^{1},x_{n}^{2},\ldots ,x_{n}^{k}\right\}$. Subsequently, the data are encoded using the polar encoding algorithm. To make the decoding algorithm resistant to noise interference in the channel, the encoded signal is usually processed with noise. The whole process is expressed as

(3) $$z_{n}={\left(x_{n}\right)}^{*}+\mu_{n},$$

where $z_{n}$ is the received codeword, $\mu_{n}$ is noise, and ${\left(\cdot \right)}^{*}$ is the codeword after encoding. The training data pair $\left(z_{n},x_{n}\right)$ is obtained through this process, where $z_{n}\in Z$ is the input of the network, and $x_{n}\in X$ is the training label.

The structure of the neural network decoder (NND) can be expressed as $\left\{z_{n},S_{1},S_{2},\ldots ,S_{L},{\hat{x}}_{n}\right\}$, where L is the number of network layers, $S_{l}$ refers to the l−th layer and 1≤l≤L, and ${\hat{x}}_{n}$ denotes the output. For each layer, there is an associated $S_{l-1}\times S_{l}$ weight matrix $w^{\left(l\right)}$ and a $1\times S_{l}$ bias vector $b^{\left(l\right)}$ [32]. The output vector of layer l is denoted as

(4) $$z_{n}^{\left(l\right)}=f_{l}\left(z_{n}^{\left(l-1\right)}\right)=\rho \left(z_{n}^{\left(l-1\right)}*w^{\left(l\right)}+b^{\left(l\right)}\right),$$

where ρ(∗) is the activation function [32]. When 1≤l≤L−1, it can be expressed as

(5) ρ(m)=max(0,m).

When l=L, it can be expressed as

(6) $$\rho \left(m\right)=\frac{1}{1+e^{-m}}.$$

It is the sigmoid function, which restricts the values of the output layer to the range (0, 1). The NND can be denoted as

(7) $$p_{n}=f\left(z_{n}\right)=f_{L}^{\rho }(f_{L-1}^{\rho }\left(\ldots (f_{1}^{\rho }\left(z_{n}\right)\right))),$$

where $p_{n}$ refers to the vector of probabilities. $f_{l}^{\rho }$ refers to the activated output of the l−th layer. Binarize $p_{n}$ to obtain ${\hat{x}}_{n}$**.**

Next, the parameters are updated utilizing backpropagation algorithm [33]. In NND, the commonly used loss function is MSE or cross-entropy (CE) [32] as

(8) $$\begin{array}{c} Loss_{MSE}=\frac{1}{K\times Z_{num}}\overset{K-1}{\underset{i=0}{\sum }}\overset{Z_{num}-1}{\underset{j=0}{\sum }}{\left(x_{j}^{i}-p_{j}^{i}\right)}^{2},\\ Loss_{CE}=-\frac{1}{K\times Z_{num}}\overset{K-1}{\underset{i=0}{\sum }}\overset{Z_{num}-1}{\underset{j=0}{\sum }}\left[\left(1-x_{j}^{i}\right)\log\left(1-p_{j}^{i}\right)+x_{j}^{i}\log\left(p_{j}^{i}\right)\right], \end{array}$$

where $Z_{num}$ and K are the mini-batch size and information bits of each codeword, respectively. $x_{j}^{i}\in x_{n}$ and $p_{j}^{i}\in p_{n}$ represents the bit value and output probability of the i−th bit in the j−th codewords.

## 3. DIR-Net Method

The proposed DIR-Net consists of two subnetworks, a denoising subnetwork, and a decoding subnetwork. The cores of both networks are the attention modules. These two parts utilize a specially designed attention structure to refine the input information gradually and complete the process of decoding in the end. We first introduce the overview processing of our polar encode/decode framework. Then, the denoising subnetwork and decoding subnetwork are introduced. In the end, we introduce the training details.

### 3.1. Overview of the Framework

**Figure 1:**
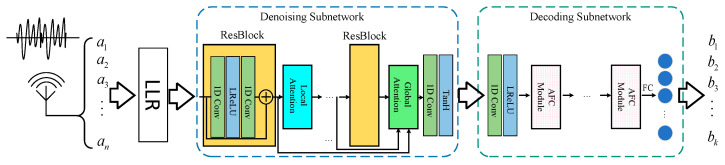
Overview of the proposed framework.

The whole framework of the proposed DIR-Net is shown in Figure 1. The input is the encoded signal transmitted through the channel, while the output is the decoded signal by DIR-Net.

The information sequence sent by the source transmitter is encoded into polar codes. Subsequently, the codeword is modulated to suit transmission in the communication channel. Usually, there is noise existing in the channel. Thus, the signal model for received symbols can be formulated as follows:

(9) $$z_{n}={\left(x_{n}\right)}^{*}+\mu_{n},$$

where $\mu_{n}\sim \left(0,\frac{\sigma^{2}}{2}\right)$ represents Gaussian white noise. The goal of our work is to predict of estimation ${\hat{x}}_{n}$ of $x_{n}$ from the received signal $z_{n}$.

As shown in Figure 1, there are three processing stages in our decoding method, the preprocessing stage, the denoising stage, and the decoding stage. The preprocessing stage is to obtain the log-likelihood ratio (LLR) values by assuming the distribution of Gaussian noise. The LLR of the transmitted codeword is

(10) $$\mathrm{LLR}\left(z_{n}\right)=\ln\frac{P\left(x=0|z_{n}\right)}{P(x=1|z_{n})}=\frac{2z_{n}}{\sigma^{2}}.$$

The denoising stage and decoding stage are performed by the two subnetworks in DIR-Net, which refine and decode the signal via cascaded attention modules. Assuming that the output is $O_{\mathrm{LLR}}=\left\{l1,l2\ldots lN\right\}$ through the LLR process, where $O_{\mathrm{LLR}}$ contains the noise superimposed in the process of channel transmission, the obtained LLR value is fed into the denoising subnetwork to perform refinement and obtain the denoising codewords M={m1,m2…mN}. After the denoising subnetwork, a step-wish decoding subnetwork with attention mechanism is utilized to decode the polar codes.

The reasons for building a two-stage decoding network are as follows: The signal will introduce much noise during transmission in the channel. This noise will bring a negative impact on decoding accuracy. So, we construct a denoising subnetwork to reduce the noise in the signal. The decoding accuracy of the decoder will be greatly improved after that.

In addition, we introduce an attention mechanism to the denoiser and decoder. The attention mechanism in the denoiser separates the valid information from the noise, which further refines the information and prepares it for decoding. The attention mechanism in the decoder allows further adaptive selects useful information in decoding process. The important information is given higher weights compared with non-important information. The accuracy can be improved by the two-stage information refinement. On basis of the above considerations, we design a more advanced network structure than existing NND methods, as shown in Figure 1.

The structure of decoder and denoiser whole work is explained in detail below.

### 3.2. Attention-Based Denoising Subnetwork

After comparing several neural networks, we choose the residual network as the backbone of the denoising subnetwork. A residual network is cascaded by some residual blocks with the same structure, and each block contains several layers. The core idea of the residual block is learning only the difference between the input and output features, which is much similar to the denoising process. With the signal with noise as input, the residual block learns to extract noise and subtracts it from the signal. On the other hand, residual network naturally has the advantages of easy training and preventing gradients vanish. For the design of the denoising subnetwork, we refer to the structure proposed in [34] which is each block contains two convolution layers and one activation layer. We connect an attention module behind each residual block. The reason is that we think the features extract by the network are not all helpful for denoising. Some features may be useless or even negative. The attention module takes the output of the residual block as input, generating a suitable weight for each feature. Multiply these weights by the corresponding features, the features will be enhanced or suppressed. For the design of the attention module, we refer to the structure proposed in CBAM [25]. A convolution layer and a global average pooling layer are used to generate spatial feature weights and channel feature weights, respectively. For the last attention module, all the features from previous residual block are involved in the weight calculation, which makes the generated weight more accurate. By feeding the weighted features to the following residual blocks, a better denoising effect will be obtained.

Usually, signal denoising relies more on local features. In other words, the denoising of each information bit is more dependent on its neighbors than distant information bits. Therefore, a convolution network is constructed to perform the denoising task, which is powerful in extracting local structural features in computer vision tasks. The structure of our denoising subnetwork is shown in Figure 1.

**Figure 2:**
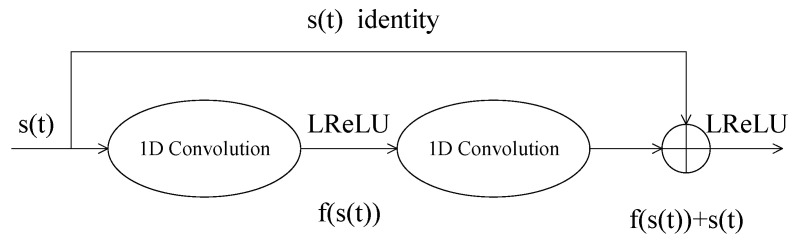
Structure of residual block.

Considering that the signal is one-dimensional, a 1D convolutional layer is utilized to construct our network. At the beginning of the network, a convolutional layer is used to map the signal to the feature space. After that, a series of residual blocks and attention modules are utilized to denoise the signal in the feature domain. The residual blocks are responsible for feature extraction, and the attention modules are responsible for separating valid information and noise. Through this cascade, signals can be gradually refined. The number of residual blocks is set using a tradeoff between denoising quality and speed. Specifically, the structure of our residual block is shown in Figure 2.

The residual block is composed of a convolution branch and a short connection branch. The convolution branch contains two 1D convolution layers and an activation layer. The output H(x) of the residual block is defined as

**Figure 3:**
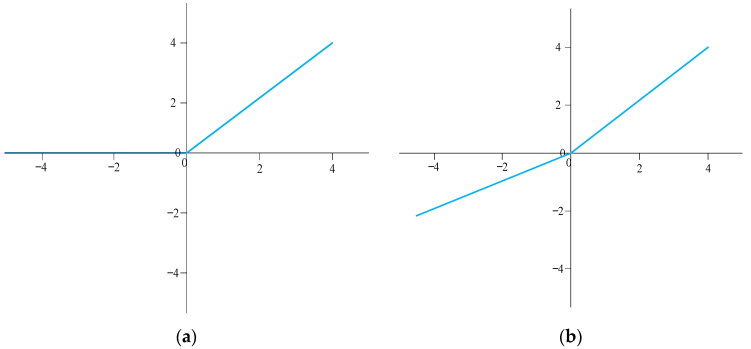
Curves of (**a**) ReLU and (**b**) LReLU.

(11) H(s(t))=s(t)+f(s(t)),

where s(t) is the input of both branches, and f(s(t)) is the output of the convolution branch. The final output is the sum of the two branches. Here, leaky ReLU (LReLU) is utilized as activation function in our network. It is defined as follows:

(12) $$LReLU(k_{i}\left(s\left(t\right)\right)=\{\begin{array}{ll} k_{i}\left(s\left(t\right)\right) & if\;k_{i}\left(s\left(t\right)\right)>0\\ a\cdot k_{i}\left(s\left(t\right)\right) & else \end{array},$$

where a is a constant value that decides the slope of the activation function on the negative semiaxis, and $k_{i}\left(s\left(t\right)\right)$ is the feature of s(t) extracted by the i-th layer of the neural network. As is shown in Figure 3, LReLU can preserve negative values that are beneficial for our task, as the range of our data is [−1, 1].

In this way, the neural network can be forced to learn the noise distribution and then remove the noise from the input signal via an addition operation. Compared with direct learning, the residual method enables the network to converge more easily and achieve a better denoising effect. We connect an attention module after each residual block. The attention module learns to suppress noise and preserves only valid information. To improve the denoising performance of the network, multiple residual blocks and attention modules are cascaded as shown in Figure 3, such that the codeword can be refined by these blocks step-by-step. Consequently, the noise of the signal reaches a relatively low level, thereby laying a foundation for the subsequent decoding processing.

We add two attention modules to the network for separating the signal and noise, namely, local and global attention modules. As an independent module, the attention module only changes the value of the feature without changing its shape. Thus, it can be inserted anywhere on the network. A local attention module is inserted between every two residual blocks to optimize local features. Furthermore, the output of the feature by the last residual block is optimized by a global attention module.

**Figure 4:**
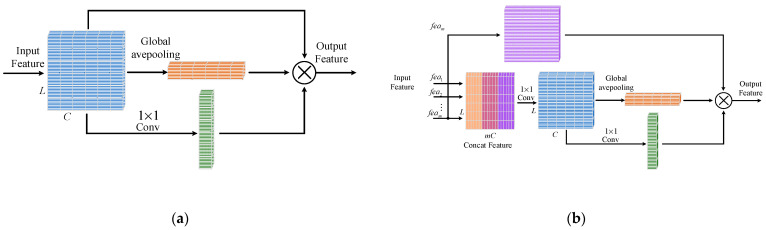
Structure of (**a**) local attention module and (**b**) global attention module.

The structure of the attention module is shown in Figure 4. For a feature with the shape C×L that needs to be processed, it needs to be divided into two branches. Here, C is the number of feature channels, and L is the length of the feature. For the first branch, a global average pooling layer is utilized and the channel feature with shape C×1 is outputted. For the other branch, an 1×1 convolution layer is used, and the spatial feature with shape 1×L is outputted. Both features are processed by the sigmoid function to limit the value to the range [0, 1]. They are multiplied with the original features as weights. Among them, the normalized channel feature is multiplied by the original feature on each channel. After that, the normalized spatial feature is multiplied by the channel-weighted features on every location in the spatial dimension. Unlike the local attention module, the attention weights of the global attention module derive from all shallow features. The features from all residual blocks are concatenated together, and a 1×1 convolution layer is utilized to generate the features that have the same shape as the final feature. The weight matrix is generated from this feature and applied to the final feature.

In the last part of the subnetwork, a convolutional layer is used to map the codewords from the feature domain to the signal domain. A TanH activation function limits the signal value to [−1, 1], which is defined as follows:

(13) $$TanH\left(x\right)=\frac{e^{x}-e^{-x}}{e^{x}+e^{-x}}.$$

The detailed values of the denoising subnetwork parameters are listed in Table 1.

In Table 1, *k* is the kernel and *s* is the stride. For example, *k*3*s*1 denotes that the kernel is 3 and stride is 1. GAP represents global average pooling.

In the training stage, the loss function (MSE) used to optimize the network is expressed as

(14) $$MSE=\frac{1}{m}\overset{m}{\underset{i=1}{\sum }}{\left(y_{i}-g\left(x_{i}\right)\right)}^{2},$$

where $y_{i}$ and $g\left(x_{i}\right)$ represent the true value and predicted value of the *i*-th sample, respectively, and m is the number of samples.

### 3.3. Attention-Based Decoding Subnetwork

After being processed by the denoising subnetwork, the signal is further decoded to obtain the original information. According to the encoding rules of polar codes, each encoded bit contains the information of most other bits. Thus, the process of polar encoding can be regarded as a global operation. However, one-dimensional convolution can only obtain local information for each operation. Although the receptive field can be increased to obtain global information by superimposing multilayer convolution, a deeper network is required, and two bits that are far apart cannot establish an effective connection by these means. Considering the above, the fully connected layer with attention mechanism (AFC) is adopted to construct the decoding sub-network.

In order to ensure that the decoding network to have sufficient decoding capacity to deal with various codewords, the network is usually designed to extract more feature than the decoding process really needs. For a codeword with length N, the number of neurons in the first layer is often greater than N. With the depth increase of the network, the number of neurons gradually decreases until information bits K. In the training process, the nonlinear operation of the network will automatically learn a decoding algorithm, while there must be some features extract from decoding subnetwork are invalid. Similar to denoising subnetwork, we connect an attention module after each fully connected layer except the last one to preserve useful information and suppress invalid information. Inspired by the structure proposed in image semantic segmentation [35] and translation tasks [36], we construct an encode/decode structure to generate weights for each feature. This structure has been proved to be effective in extracting semantic information. Here, we simplify it to three layers of fully connected layers, in which the number of neurons in the middle layer is less than that in the front and back layers.

**Figure 5:**
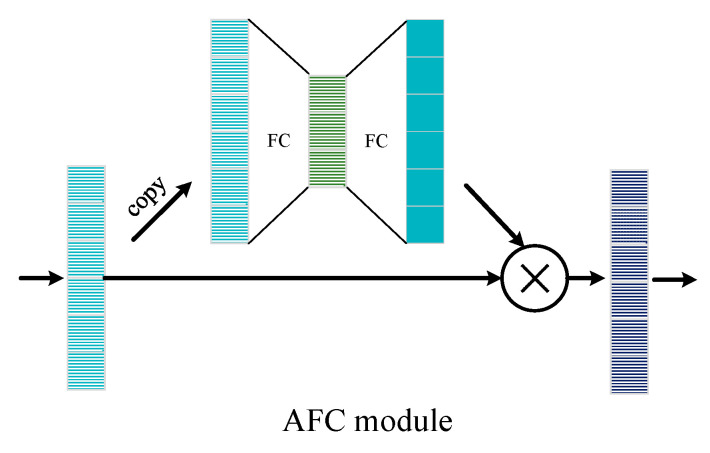
Structure of attention fully connected layer (AFC) module.

As shown in Figure 1, the decoding subnetwork is composed of three parts, the feature extraction part, the feature mapping part, and the classification part. As with the denoising subnetwork, a convolution layer is utilized to transform the input data to the feature domain. After that, the features are fed to a series of feature mapping networks composed of AFC units, which map the codeword from the polar encoding feature space to the original codeword feature space. The structure of the AFC is shown in Figure 5. The input features are first copied out and used to calculate the weights of each neuron. The weight calculation subnetwork has an encode/decode structure. The input is first squeezed by a fully connected layer to extract semantic features. After that, an expended fully connected layer is utilized to inflate the feature to its original shape. To limit the range of weights, the sigmoid function is used to normalize the feature value to [0, 1]. In the end, the normalized weights are multiplied by the original features to finish the refinement of the input feature. Through the units, this system can carry out adaptive processing on the input information according to importance, giving more weight to important information and less weight to unimportant or negative information.

In the decode subnetwork, multiple AFC units are cascaded into a multilayer AFC network to decode the input data. The output of each AFC unit serves as the input of the next layer of the AFC unit. In the process of decoding each bit, the contribution of different bits is different. The attention mechanism in the AFC unit can impose an importance coefficient on each bit self-adaptively, thereby ensuring that important information can be fully utilized, while reducing the interference of other information to the decoding process. By this means, the features can be refined step-by-step. The detailed values of network parameters of the decoding subnetwork are listed in Table 2.

Due to the inconsistent length of the codeword before and after decoding, a fully connected layer is added at the end of the decoding subnetwork. In this way, the features can be mapped to the original codewords to complete the entire decoding process. In the decoding network training phase, the decoding process is treated as a multilabel binary classification task. Accordingly, the loss function of the subnetwork is expressed as follows:

(15) $$Loss=-\left(y\cdot \log(\overset{\wedge }{y})\right)+\left(1-y\right)\cdot \log(1-\overset{\wedge }{y})),$$

where y is the real decoded data, and $\overset{\wedge }{y}$ is the predicted decoded data.

## 4. Experiments and Results

### 4.1. Experiment Setting and Training Details

To simulate and verify the effectiveness of the decoding model, the program was written in Python language, and the network part was implemented in the Pytorch deep learning framework. The computer used an Ubuntu 16 operating system.

We tested the DIR-Net method with five lengths of original codewords: 8, 16, 32, 64, and 128 code lengths, with the corresponding polar decode length of 16, 32, 64, 128, and 256, respectively. As for the training data, all the original codewords were generated randomly with the binary model. Then, they were encoded into polar codes for network training. In all training stages, the learning rate of these networks was set to 0.001 during training. To prevent the network from overfitting at the initial stage of training, the warmup training strategy was utilized. In the first 1000 iterations, the learning rate was gradually increased from 0.0001 to 0.001, while, in the subsequent training process, the learning rate remained constant until the model converged.

The neural network training process we designed was divided into three steps. Firstly, only the denoising subnetwork was trained. Here, the input to the network was the received data processed by LLR, while the trained label was the data input to the channel. Furthermore, the label data contained no noise and attenuation via the channel. Secondly, after training the denoising subnetwork, the parameters of the network were fixed, and the decoding subnetwork was trained. At this point, the output of the denoising subnetwork was regarded as the input of the decoding subnetwork, and the original unencoded codeword was taken as the label to train the decoding subnetwork. Finally, the entire network was finetuned together. At this stage, the denoising subnetwork and the decoding subnetwork were released for training at the same time, so that the two subnetworks could better work together.

### 4.2. Ablation Experiments

#### 4.2.1. Denoiser Performance

**Figure 6:**
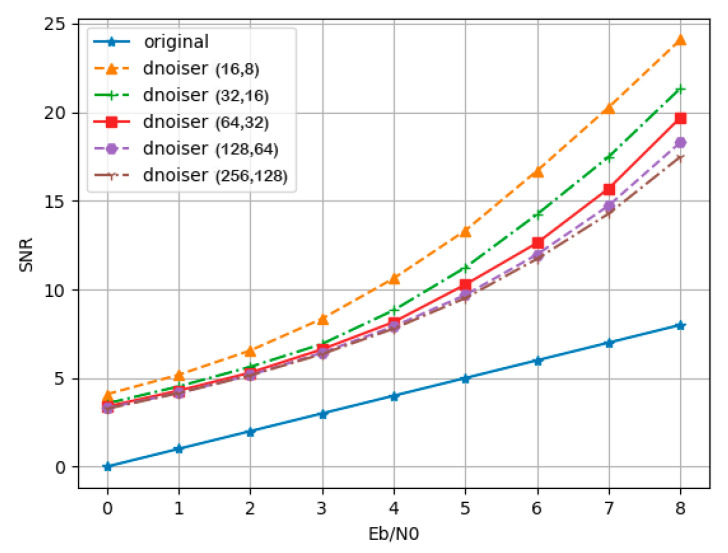
The SNR comparison of signals before and after denoising with different code lengths.

As is known, denoising is the preprocessing for codewords before decoding. The noise interference can be effectively reduced by a denoiser, which is convenient for more accurate performance of the decoder. In this section, we explore the effect of the proposed denoisers with different code lengths, and we compare the results with the absence of denoising. The denoiser is composed of a convolution layer, which can denoise codes with different lengths without changing the structure. As a result, (16, 8) polar codes were adopted as training data, and (16, 8), (32, 16), (64, 32), (128, 64), and (256, 128) polar codes were utilized as test data in the experiment. The test signal-to-noise ratio (SNR) ranged from 0 dB to 8 dB for different code lengths. The results before and after denoising are shown in Figure 6.

In Figure 6, the blue line is the SNR of the signal received without denoising. As shown, the convolutional denoiser utilized could achieve denoising for different code lengths. Our denoising network was trained at the code length of (16, 8); thus, the optimal effect was achieved for this code length as we expected. Furthermore, the data with other code lengths were fed into the network for testing. It can be seen in Figure 6 that, even though the denoiser was not trained on these code lengths, the noise could still be removed effectively, mainly benefiting from the local information processing capacity of the convolutional network. Moreover, the denoising effect was obvious when the code length was short. With the increase in code length, although the denoising ability of the denoiser weakened, it could still play a role in noise reduction. The experiment illustrates that the convolutional denoiser we utilized could denoise the received codeword information effectively such that the output result of the decoder was less affected by noise and more accurate.

**Figure 7:**
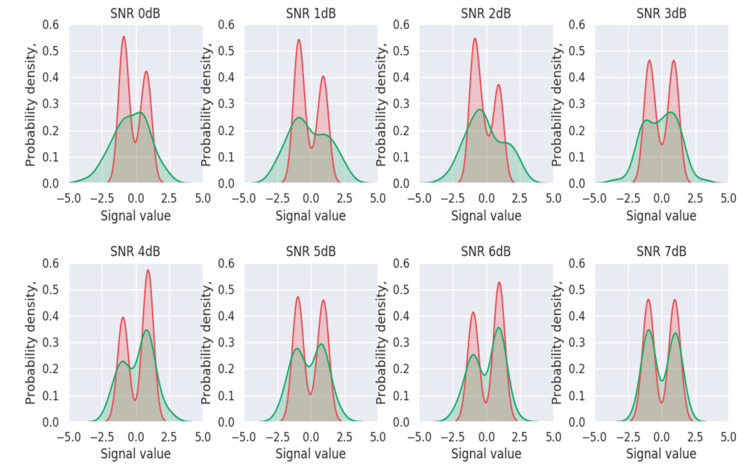
Probability density function with and without denoiser under different signal-to-noise ratios.

The probability density distribution obtained after training with 0 dB of SNR is shown in Figure 7. The green area is the signal not denoised by the denoiser, while the pink area is the signal denoised by the denoiser. When the SNR was small, the codeword information suffered much noise interference. It can be seen from the green area that the signal was submerged by noise. However, after passing through the denoiser, more noise could be removed and the BPSK modulated signal was obtained, indicating that the denoiser could work effectively.

#### 4.2.2. AFC Module Number

The decoder is the core part of the DIR-Net algorithm, while the AFC is the significant unit that constructs the decoder, for which the number of modules plays a crucial role in the performance of the decoding. In this section, we investigate the impact of the number of AFC modules on the performance of the DIR-Net algorithm. In the experiment, (16, 8) polar codes were selected as training and test data, i.e., the code length *N* = 16, and the information bit length *K* = 8. In addition, Gaussian white noise was added to the encoded data as input of the network. At the same time, the corresponding original codeword was used as the training label. The network structure in our experiment was the same as that introduced in the previous section, consisting of a denoiser and a decoder. The denoiser was an attention-based residual network, and the decoder was composed of a series of AFC units.

**Figure 8:**
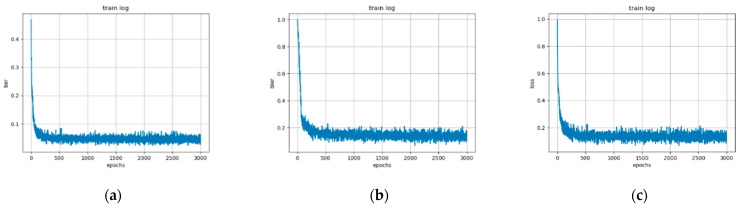
The (**a**) BER, (**b**) BLER, and (**c**) loss curves with epoch.

In our experiment, five network structures were adopted for comparison where the only difference among them was the number of AFC units in the decoder. Thus, 1–5 AFC units were utilized to investigate the impact of AFC module number on decoding performance. In the training process, all $2^{K}$ codewords were involved in training. Each network structure was trained until convergence. The BER, BLER, and loss curves of five AFC modules converged with the number of iterations increasing, as shown in Figure 8. It is obvious that the network was finally convergent. When the AFC number was less than five, the network converged more quickly.

**Figure 9:**
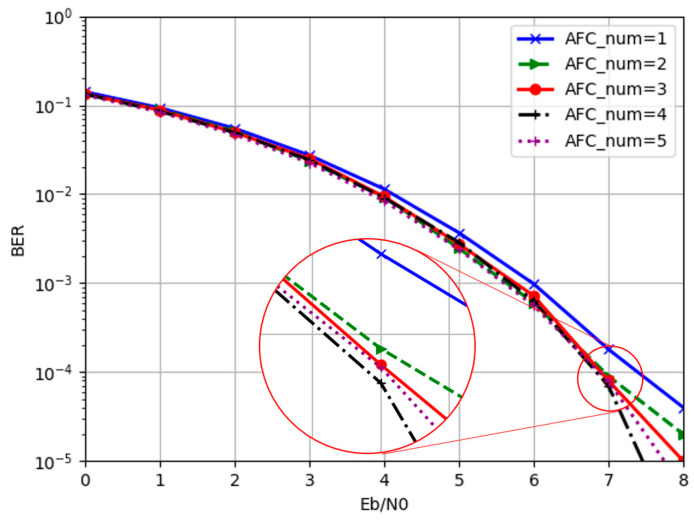
The comparison of BER performance with different AFC numbers.

The effects of different AFC module numbers are shown in Figure 9. When the number of AFC units was one, its bit error rate was still relatively high although the network could complete the task of decoding. Especially in the case of high SNR, compared with other network structures, the decoding performance still needed to be improved. The network performance tended to be saturated when the number of AFC units increased to four, and the decoding performance improvement gradually became smaller. When the number of AFC units increased to five, the decoding performance deteriorated. Comparing the bit error rate curves of four and five AFC units, it can be seen that, when the signal-to-noise ratio was less than 7 dB, the two were very close. The advantages of the decoder network depth began to appear when the signal-to-noise ratio was located at 7–8 dB. In addition, the performance gap of each network gradually increased with the SNR, indicating that the deep network had better generalization capabilities.

#### 4.2.3. Training Rate

**Figure 10:**
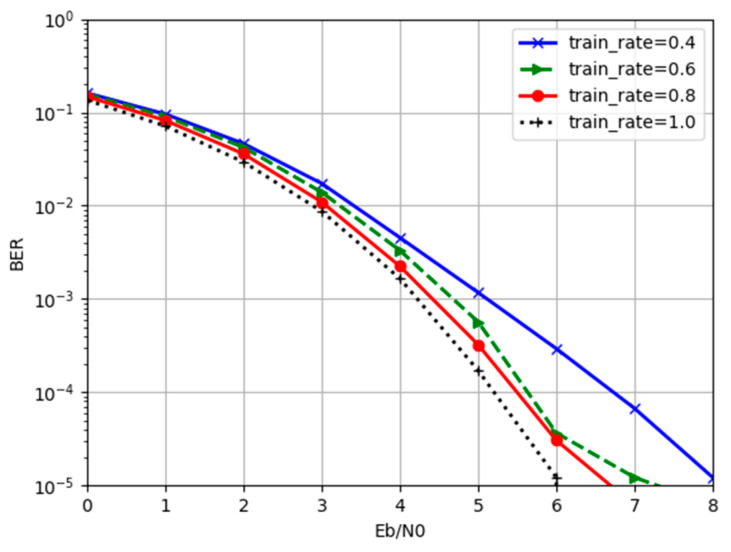
The BER performance comparison for different training rates.

In this section, we investigate the impact of the amount of data in the training set on the DIR-Net decoding algorithm. There are a total of $2^{K}$ codewords when the information bit length is *K*. In theory, when $2^{K}$ codewords are involved in training, the decoding network obtains the best decoding performance. When the value of *K* is small, it is feasible to use full codeword training. However, as the length of the codewords increases, the number of corresponding codewords increases exponentially. Thus, it is impossible for all codewords to participate in training. During the training process, we only selected part of the data as the training set. Specifically, we calculated the total amount of data participating in training as follows:

(16) $$n_{d}=r_{d}\times 2^{K},$$

where $r_{d}$ is the data utilization rate with a value range of (0, 1], representing the ratio of the number of codewords participating in training to the total number of codewords. In our experiment, the number of information bits of our codeword *K* was 16, and the encoded codeword bit *N* was 32. In contrast, we set $r_{d}$ to 0.4, 0.6, 0.8, and 1.0 to train the network. During the test phase, a batch of codewords was extracted randomly into the network for decoding. The SNR/BER curve of the obtained decoder is shown in Figure 10.

As can be seen from Figure 10, even without using all codewords for training, the trained decoding algorithm could still decode codewords that had not been seen before. This indicates that the decoding algorithm learned the rules of codewords rather than simply memorizing the correspondence of codewords. On the other hand, as the value of $r_{d}$ increased, the performance of the decoding network also improved. This is because the decoding algorithm recognized a more diverse combination of codewords and noise in the decoding process. The generalization and robustness of the denoiser and decoder were improved.

### 4.3. Comparison with Other Methods

#### 4.3.1. Comparison Based on BER

**Figure 11:**
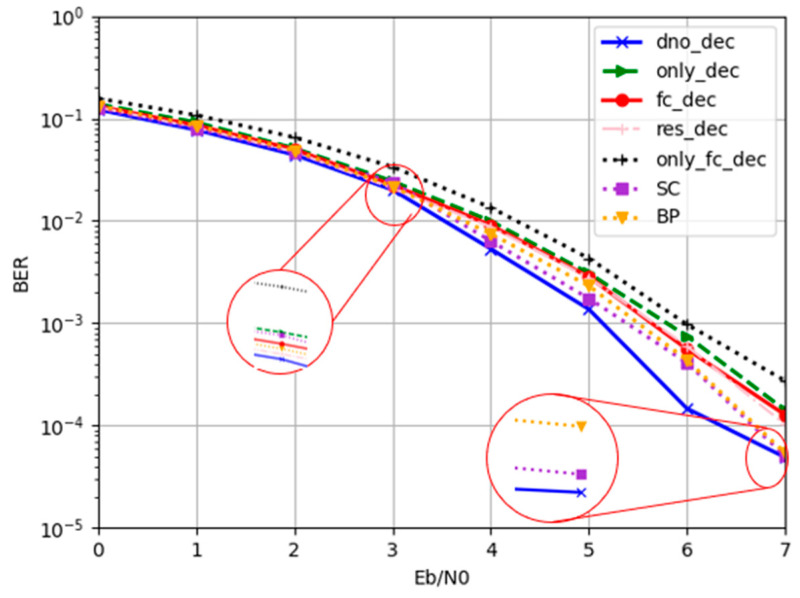
BER performance comparison of different network structures and traditional algorithms.

In this section, (32, 16) polar codes were selected to evaluate DIR-Net. We compared this network with other polar decode methods based on deep learning. In the process of testing, some codewords were generated randomly, and encoding was performed according to the polar encoding rules. To simulate the transmission of information in the channel, different levels of Gaussian white noise were added to the encoded codewords. Then, the codewords were taken as test data with diverse deep learning methods. Among the methods, the DIR-Net method with attention-based denoising and attention-based decoding was denoted as “dno_dec”. In order to evaluate the decoder performance, we used the DIR-Net method without denoiser as the comparison, which is denoted as “only_dec”, whereas “only_fc_dec” denotes the fully connected decoder proposed in [27], which does not contain a denoiser, and “fc_dec” denotes the addition of the attention-based denoiser in our paper to [27]. To further evaluate the performance of the attention-based denoiser and decoder, we compared it with the residual decoder, which was proposed in [29], denoted as “res_dec”. We additionally compared DIR-Net with the traditional SC and BP algorithms to comprehensively evaluate the performance. We tested the methods at different signal-to-noise ratios, SNR = [0, 1, 2, 3, 4, 5, 6, 7, 8] dB, and the curves are shown in Figure 11.

Firstly, we compared only_dec and only_fc_dec, which contained only the decoding network without the denoising subnetwork. As shown in Figure 11, when there was only the decoder, the decoding performance of the AFC decoder was significantly better than that of the fully connected decoder with the same number of layers. This illustrates that the attention mechanism in the AFC units indeed improved the decoding performance of the polar codes. On the other hand, we found that, after adding denoising subnetworks to the decoding algorithm, i.e., “dno_dec” and “fc_dec”, the decoding performance of the network was significantly improved. Meanwhile, we also compared the “dno_dec” algorithm with “rec_dec”, and it can be seen in Figure 11 that the DIR-Net method had better decoding performance due to the addition of the attention mechanism in both the denoiser and the decoder. In addition, the proposed method had a lower BER compared to the conventional SC and BP algorithms.

**Figure 12:**
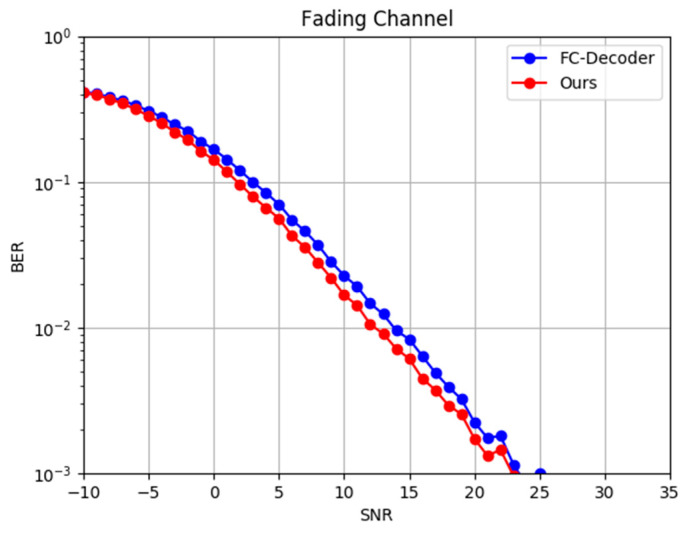
BER performance of FC-Decoder and DIR-Net in a Rayleigh fading channel.

In the real communication process, fading is often present in the channel. To evaluate the performance of the DIR-Net method in fading channels, Rayleigh fading was taken as an example to perform a simulation. The same dataset was utilized to train and test the fully connected deep learning decoding method proposed in [28], which was specifically designed to decode polar codes in a Rayleigh fading channel. The BER/SNR curves of the two methods are shown in Figure 12.

It can be seen in Figure 12 that, after adding Rayleigh fading, the difficulty of decoding was significantly increased. As a result, the bit error rate of the decoding algorithm was much higher compared to when only Gaussian noise was introduced. With the addition of the same Rayleigh fading condition, the decoding performance of the DIR-Net method was significantly better than that of the FC-Decoder, which demonstrates that our proposed DIR-Net may be a strong candidate for resisting Rayleigh fading due to its lower BER.

#### 4.3.2. Comparison Based on Decoding Time

We compared DIR-Net with the traditional method in terms of BER, but also in terms of decoding time. In this experiment, we used Windows 10 system with 16G of running memory and an NVIDIA GeForce RTX3050Ti Laptop GPU as the test environment.

In order to compare the speed of each decoding technique more comprehensively, we test the denoiser speed, decoder speed, and denoiser-decoder speed of DIR-Net method with different code lengths, including (16, 8), (32, 16), (64, 32), (128, 64), and (256, 128) polar codes. To further evaluate the speed of DIR-Net, it is compared with traditional SC algorithms, BP algorithms, and deep learning-based methods only_fc_dec [27], FC_Decoder [28], and res_dec [29]. The decoding time for each method listed in Table 3.

Benefiting from the parallel ability of deep learning algorithms and GPU, the deep learning approach outperforms the SC and BP algorithms in terms of running speed, as shown in Table 3. Additionally, the decoding speed is little affected with the code length increases.

When utilizing only the decoder with the attention mechanism, the DIR-Net technique has a decoding comparable speed with that of the only_fc_dec and the FC-Decoder method. Since the attention mechanism for deep feature extraction is used in both the denoiser and decoder, we gain a significant improvement in decoding accuracy in exchange for a slight loss in speed.

## 5. Conclusions

In this paper, a novel polar decoding algorithm, DIR-Net, based on deep learning was proposed. To achieve the goal of separating effective information from interference information, we carefully designed several information optimization modules based on the attention mechanism. Furthermore, we constructed a high-performance decoding network based on the proposed attention modules. The proposed network contains two subnetworks with different functions, i.e., the denoising subnetwork to remove the noise brought in the transmission channel and the decoding subnetwork to decode the denoised information. Benefiting from the cascaded attention module, the information can be refined step-by-step, thus obtaining a low error rate. Furthermore, the residual connections in the denoising subnetwork facilitate the convergence of this network. In the end, a series of experiments were conducted to verify the effectiveness of the DIR-Net algorithm. The experimental results show that our method had a low BER in the presence of both noise and fading in the channel. Compared with existing decoding methods, DIR-Net achieved better performance.

## Figures and Tables

**Table 1:** Network structure of denoising subnetwork.

Block	Layer		Number
Input	*N* × 1		× 1
ResBlock	Conv + LReLU *k*3*s*1 *N* × 16 Conv *k*3*s*1 *N* × 16 Add + LReLU —— *N* × 16		× 4
Local Attention	GAP + Sigmoid—1 × 16	Conv + Sigmoid *k*1*s*1 *N* × 1	
	Multiply —— *N* × 16		
ResBlock	Conv + LReLU *k*3*s*1 *N* × 16 Conv *k*3*s*1 *N* × 16 Add + LReLU —— *N* × 16		× 1
Global Attention	Concat —— *N* × 64 Conv + LReLU *k*1*s*1 *N* × 16 LReLU —— *N* × 16		
	GAP + Sigmoid—1 × 16	Conv + Sigmoid *k*1*s*1 *N* × 1	
	Multiply —— *N* × 16		
Output	Conv + Tanh *k*3*s*1 *N* × 1		× 1

**Table 2:** Network structure of decoding subnetwork.

Block	Layer	Number
Input	*N* × 1	× 1
ConvBlock	Conv + LReLU *k*3*s*1 *N* × 16	× 1
AFC Module	FC + LReLU 512 × 1 FC + LReLU 256 × 1 FC + Sigmoid 512 × 1 Multiply 512 × 1	× 1
AFC Module	FC + LReLU 256 × 1 FC + LReLU 128 × 1 FC + Sigmoid 256 × 1 Multiply 256 × 1	× 1
AFC Module	FC + LReLU 128 × 1 FC + LReLU 64 × 1 FC + LSigmoid 128 × 1 Multiply 128 × 1	× 1
AFC Module	FC + LReLU 64 × 1 FC + LReLU 32 × 1 FC + Sigmoid 64 × 1 Multiply 64 × 1	× 1
Output	FC K × 1	× 1

**Table 3:** Comparison of DIR-Net Decoding Time and Conventional Techniques with Different Code Lengths.

	16-8	32-16	64-32	128-64	256-16
Denoiser	8.310 × 10^−4^	8.386 × 10^−4^	8.397 × 10^−4^	8.414 × 10^−4^	8.456 × 10^−4^
Decoder	3.371 × 10^−4^	3.386 × 10^−4^	3.409 × 10^−4^	3.423 × 10^−4^	3.431 × 10^−4^
DIR-Net	1.168 × 10^−3^	1.180 × 10^−3^	1.181 × 10^−3^	1.183 × 10^−3^	1.187 × 10^−3^
res_dec	7.089 × 10^−4^	7.115 × 10^−4^	7.120 × 10^−4^	7.145 × 10^−4^	7.469 × 10^−4^
only_fc_dec	1.844 × 10^−4^	1.856 × 10^−4^	1.857 × 10^−4^	1.864 × 10^−4^	1.868 × 10^−4^
FC_Decoder	1.818 × 10^−4^	1.819 × 10^−4^	1.836 × 10^−4^	1.840 × 10^−4^	1.848 × 10^−4^
SC	3.039 × 10^−3^	9.498 × 10^−3^	3.122 × 10^−2^	1.106 × 10^−1^	1.408 × 10^−1^
BP	3.727 × 10^−2^	9.214 × 10^−2^	2.189 × 10^−1^	5.047 × 10^−1^	1.1512

## Data Availability

Not applicable.
